# Factors associated with consultation behaviour for primary symptoms potentially indicating colorectal cancer: A cross-sectional study on response to symptoms

**DOI:** 10.1186/1471-230X-12-100

**Published:** 2012-08-03

**Authors:** Ryan J Courtney, Christine L Paul, Robert W Sanson-Fisher, Finlay A Macrae, John Attia, Mark McEvoy

**Affiliations:** 1 The Priority Research Centre for Health Behaviour, School of Medicine and Public Health, Faculty of Health, The University of Newcastle, Newcastle, Australia; 2 Hunter Medical Research Institute, Newcastle, NSW, Australia; 3 Department of Colorectal Medicine and Genetics, The Royal Melbourne Hospital, Melbourne, Australia; 4 The Centre for Clinical Epidemiology and Biostatistics, Faculty of Health, The University of Newcastle, Newcastle, Australia

## Abstract

**Background:**

Little data exists on the factors associated with health care seeking behaviour for primary symptoms of colorectal cancer (CRC). This study aimed to identify individual, provider and psychosocial factors associated with (i) ever seeking medical advice and (ii) seeking *early* medical advice for primary symptoms of colorectal cancer (CRC).

**Methods:**

1592 persons aged 56–88 years randomly selected from the Hunter Community Study (HCS) were sent a questionnaire.

**Results:**

Males and those who had received screening advice from a doctor were at significantly higher odds of ever seeking medical advice for rectal bleeding. Persons who had private health coverage, consulted a doctor because the ‘symptom was serious’, or who did not wait to consult a doctor for another reason were at significantly higher odds of seeking *early* medical advice (< 2 weeks). For change in bowel habit, persons with lower income, within the healthy weight range, or who had discussed their family history of CRC irrespective of whether informed of ‘increased risk’ were at significantly higher odds of ever seeking medical advice. Persons frequenting their GP less often and seeing their doctor because the symptom persisted were at significantly higher odds of seeking *early* medical advice (< 2 weeks).

**Conclusions:**

The seriousness of symptoms, importance of early detection, and prompt consultation must be articulated in health messages to at-risk persons. This study identified modifiable factors, both individual and provider-related to consultation behaviour. Effective health promotion efforts must heed these factors and target sub-groups less likely to seek early medical advice.

## Background

### Colorectal cancer (CRC): the burden of illness

Internationally, CRC is diagnosed in over one million persons annually (9.4% of all cancer diagnoses) and ranks as the fourth leading cause of cancer related death [1]. On average, 50% of CRC cases are living five years following diagnosis [2-4]. Survival rates for CRC are inversely related to stage at diagnosis with early staged-localised CRC 5-year survival rate at 90% compared to approximately 10% for distant metastatic CRC [5]. The rate of early detection is relatively low with approximately 40% of CRC patients diagnosed at a localised stage [5].

### Primary symptoms and clinical presentation of CRC

Rectal bleeding and change in bowel habit are common potential symptoms of CRC experienced in the population [6-9]. Past studies have estimated 37-84% of all CRCs present with rectal bleeding [10-12]. Change in bowel habit, broadly termed as diarrhoea or constipation, generally refers to a change in frequency of defecation, consistency of stool, shape of stool or difficulty in evacuation [13]. Change in bowel habit is the symptom most associated with patient delay in presentation [14] and presents in approximately 48-77% of CRCs, with increased frequency of defecation/diarrhoea accounting for the largest proportion of cases [10-12].

### The paradoxical relationships among symptom duration, colorectal cancer staging and survival

The paradoxical relationship between symptom duration, CRC staging and survival continues to be debated in the literature [10,15-17]. Intuitively, a reduction in diagnostic and therapeutic delay should be accompanied by an improved survival rate [17]. This has been supported for breast cancer, with delays of 3–6 months associated with poorer survival [18], however, for CRC, it is axiomatic that reduced diagnostic and treatment delay will be accompanied by earlier-stage detection and improved prognosis [19]. Several studies have indicated that symptom duration is unrelated to pathological stage at diagnosis [10,20,21]. A potential explanation for this paradoxical finding may be the limited potential benefit of early detection and treatment for those persons with particularly aggressive tumour biology [22]. Conversely, other studies have indicated that patients with symptom duration equal to or greater than 3 months are less likely to have Stage 1 tumours [23,24]. Although inconclusive, other studies have indicated an inverse relationship, whereby a shorter symptom duration period is associated with poorer staging and survival [25,26].

It is important however that the necessity of prompt medical advice seeking is not forgotten especially given that only a minority of CRC cases are detected asymptomatically through screening, between 5-20% of all cases [14,27]. While the pattern of presentation is slowly changing towards more CRCs being identified through screening, for the foreseeable future, the high rate of symptomatic presentation to the health care system is likely to continue [28,29]. Most commonly the first step towards diagnosis is via symptomatic presentation to primary care [14,28] with approximately a quarter of symptomatic cases presenting as an emergency [28-31], usually with bowel obstruction [32]. Studies examining emergency presentation to hospital departments have indicated a median symptom duration of three months prior to admission, without patients necessarily presenting at any earlier point to primary care [33,34]. For cases presenting to emergency, the mortality rate [35] and five year-survival rate is poor [36-38], likely because of the serious state of illness across multiple organ systems that patients develop after obstruction occurs. A recent UK National Health Service review of the “Two Weeks” program indicated that reduction in delay can have the important patient outcome of minimising complications, e.g. bowel obstruction, which may have an effect on survival [39]. Ideally, a large public health gain may be achieved for the late-presenting patient group if medical intervention occurs at an earlier stage and the proportion of emergency admitted cases is decreased [40].

### Bridging the evidence practice gap

Our understanding of the factors associated with medical advice seeking behaviour for primary symptoms potentially indicating CRC is largely restricted to a few community and population-based studies, predominantly focusing on rectal bleeding. An Australian community-based study indicated that divorced, separated or retired persons were more likely to *ever* consult a doctor for rectal bleeding [6]. Other international studies have identified that the following predict *ever* seeking care for CRC symptoms in the community setting: older age (> 45 years), being a non-smoker, being employed, having constipation rather than diarrhoea, and symptom specific characteristics such as greater concern, severity, and frequency of symptoms [41,42]*.* In relation to early medical advice seeking, the literature has largely focused on those delaying greater than three months [6,43]. Previous research suggests that adults experiencing rectal bleeding delay or fail to consult a doctor due to a perception that the condition is not serious [6,7,43]. Identified *triggers* for seeking medical advice for rectal bleeding include: greater perceived seriousness, persistence and/or nuisance of the symptom, pain or discomfort, opportunity during an existing consultation to discuss the symptom, and pressure from a relative [44,45]. Previous literature relating to CRC patients health care seeking behaviour have also highlighted other key factors associated with patient delay including: patient appraisal, recognition and knowledge of symptoms, symptom characteristics, and emotional response to symptoms [46-50].

Little is known about medical advice seeking for change in bowel habit, which is limited to a handful of studies more generally examining medical consultation rather than time taken to seek medical advice [41,51,52]. The current study seeks to identify the factors associated with medical advice seeking behaviour for primary symptoms of CRC. Identification of population or individual characteristics associated with earlier medical consultation is important in shaping future public-health messages aimed at encouraging prompt medical advice seeking in the at-risk community. Therefore, this study retrospectively examined socio-demographic, provider and psychosocial factors associated with the following practices among an at-risk (56–88 years of age) cohort of community dwelling persons:

1. Ever seeking medical advice for rectal bleeding and change in bowel habit.

2. Early medical advice seeking for rectal bleeding (< 2 weeks) and change in bowel habit (< 4 weeks).

## Methods

### Design and study population

The Hunter Community Study (HCS) is a longitudinal cohort of community dwelling men and women aged 55–85 years at baseline from the Hunter Region, NSW, Australia [53]. Participants were randomly selected from the NSW State electoral roll between December 2004 and 2007. The HCS cohort provides a population profile reflecting that of the Hunter Region, state and national Australian profiles for gender and marital status but is slightly younger in age [53]. A randomly selected subsample of HCS participants (n = 1592) aged between 56 and 88 years at time of survey (November, 2009) were mailed a pen and paper questionnaire. A reminder telephone call was made to non-responders at 4–6 weeks following initial mail out.

### Questionnaire

Respondents were asked separately whether they had ever experienced either rectal bleeding or a persistent change in bowel habit (diarrhoea/constipation) that lasted longer than two weeks. These items were used to determine the proportion of respondents ever experiencing each symptom. The item concerning rectal bleeding had been used in two previous Australian studies [6,43] while the change in bowel habit item was devised for this study. Details regarding rates of medical advice seeking are provided elsewhere [54]. Respondents indicating they had ever experienced either symptom were asked whether they had ever seen a doctor about that particular symptom*.* Response to this question, for both symptoms was used to identify the proportion who had ever sought medical advice from a doctor. Respondents indicating they had ever consulted a doctor were asked if they had noticed this symptom for the first time in the previous five years. To reduce recall bias only respondents experiencing their first symptom episode in the last five years and consulting a doctor were included in the analyses of early medical advice seeking. Such respondents were asked to specify the time taken to seek medical advice and trigger(s) for seeking medical advice. The response options relating to triggers for seeking medical advice were forced choice with an “Other (Please specify)” option included. For rectal bleeding, location of bleeding, colour, frequency and level of concern were assessed. For change in bowel habit, type of irregular bowel movement, level of discomfort/pain, and frequency of symptom were assessed.

All respondents were asked about their family history of CRC and age at diagnosis across first and second degree relatives. Respondents answers to questions relating to doctor screening of family history of CRC and identification of any possible ‘increased risk’ were used to derive a *family history of CRC discussed with doctor* variable with three levels (never discussed, discussed and informed of possible ‘increased risk’, discussed and not informed of possible ‘increased risk’). Respondents were also asked to indicate whether a doctor or other health professional had ever provided CRC screening advice. 'An additional questionnaire file shows in more detail questions and response options used in this study [see Additional file 1].

### Predictors

Based on existing literature relating to the barriers and facilitators of medical advice seeking, an *a priori* investigation of the following items selected from the HCS databank were assessed: *Socio-demographic and lifestyle characteristics*, i.e. age, gender, education, marital status, country of birth, household income, retirement, private health insurance status, tobacco or alcohol use; *Clinical characteristics* i.e. general practice visits per year, previous cancer diagnosis (excluding CRC), body mass index, and co-morbidity (e.g. high cholesterol, hypertension, asthma, diabetes); and *Psychosocial characteristics i.e.* physical health, assessed using the physical health component summary score (PCS) on the short form health survey (SF-36) [55] and mental health, assessed using the Kessler Psychological Distress Scale (K-10) [56]. The PCS is a physical health summary score aggregated from the physical functioning, role-physical, bodily pain and general-health scales on the SF-36 [57]. Predictors ascertained from respondents at the time of survey completion included: trigger for seeking medical advice, symptom characteristics, first degree relative diagnosed with CRC, family history of CRC discussed with doctor, and ever received screening advice from doctor.

### Statistical analyses

Logistic regression analysis was used to determine independent factors associated with ever seeking medical advice (never consulted, consulted) and early medical advice seeking (< 2 weeks for rectal bleeding and < 4 weeks for change in bowel habit). Variables with a *p* < .25 following simple regression analysis (see Appendix) were considered for multiple logistic regression modelling (both forward and backward stepwise elimination were used to check consistency of results). Variables that met the significance cut point of *p* < .05 were retained in the final model. Data were analysed using STATA 11 (STATA, Texas, USA).

### Ethics approval

The University of Newcastle in partnership with the Hunter New England Population Health Human Research Ethics Committee granted ethical approval (H-820-0504).

## Results

### Sample demographics

Of the 1592 mailed surveys, 1117 respondents completed and returned a survey (consent rate = 70%). Respondents previously diagnosed with colorectal carcinoma (n = 24) or reporting they had undergone major abdominal surgery (n = 8) were excluded from analysis, leaving a total sample of 1085 eligible participants for analysis. For participants diagnosed with CRC or having undergone abdominal surgery information relating to the date of CRC diagnosis/abdominal surgery was not obtained. The timing of such an event (before or after) symptom episode is critical to understanding health care seeking behaviour for such respondents. To eliminate the potential for bias in study results persons diagnosed with CRC or having undergone abdominal surgery were excluded. Demographic characteristics of the sample are presented in Table 1.

**Table 1:** Demographic characteristics of study respondents (n = 1085)

	***n***	**%**
Gender		
Male	508	47
Female	577	53
Age (years)		
56-64	455	42
65-74	382	36
75-88	237	22
Country of Birth		
Australia	885	89
Other	111	11
Marital status		
In a relationship	805	77
Not in relationship	240	23
Annual household income before tax ($)		
<= 39, 999	574	58
40, 000 – 69, 999	216	22
> = 70,000	197	20
Highest Level of Education		
Secondary schooling (not-completed)	229	22
Secondary schooling (completed)	241	23
Trade qualification or TAFE:	264	25
University or other tertiary study	256	25
Other or not applicable	53	5

* Percentage of responses (excluding any missing values).

### Rectal bleeding

Of the 1075 respondents to the rectal bleeding question, 332 (31%) reported ever experiencing this symptom with 60 (18%) respondents never having consulted a doctor. Additional file 2 presents the univariate (Pearson *χ*^2^) associations between socio-demographic, clinical and psychosocial characteristics and ever seeking medical advice for rectal bleeding. Multiple logistic regression modelling identified the following significant predictors of ever seeking medical advice for rectal bleeding: being male and persons that had ever received screening advice from a doctor or other health professional (See Table 2).

**Table 2:** Multiple logistic regression analysis of factors associated with ever seeking medical advice for rectal bleeding

	**OR (95% CI)**	***p* value**
Gender		
Male	1	
Female	.51 (.26, .98)	.045
Screening advice from doctor		
Yes	4.45 (1.90, 10.41)	.001
No	1	

OR, odds ratio; CI, confidence interval.

Of the 332 respondents ever experiencing rectal bleeding, 30% (101/332) had experienced their first symptom episode in the previous five years and consulted a doctor. These respondents were included in the analyses for early medical advice seeking, in which 67% of persons had consulted a doctor within two weeks. Multiple logistic regression modelling (see Table 3) identified that early medical advice seeking (< 2 weeks) was significantly associated with private health coverage and participant trigger for seeking medical advice - ‘Thought the symptom was serious’. Persons indicating the prompting factor for consultation was “Opportunity to talk during doctor visit for other reason” were less likely to seek early medical advice.

**Table 3:** Multiple logistic regression analysis of factors associated with early medical advice seeking for rectal bleeding

	**OR (95% CI)**	***p* value**
Private health insurance		
No coverage	1	
Coverage	3.96 (1.11, 14.19)	.034
Prompt for medical consultation.		
‘Thought the symptom was serious’	5.88 (1.48, 23.30)	.012
‘Opportunity to talk during doctor visit for other reason	.15 (.04, .52)	.003

OR, odds ratio; CI, confidence interval.

### Change in bowel habit

For change in bowel habit, 1049 respondents answered this question; 195 (19%) reported ever experiencing this symptom, of which 39 (20%) respondents never consulted a doctor. Following multiple logistic regression modelling ever seeking medical advice for change in bowel habit was significantly more likely for persons: who had discussed their family history of CRC, irrespective of whether they were informed of possible ‘increased risk’ or not, with a lower household income, and who were within the healthy BMI weight range (See Table 4).

**Table 4:** Multiple logistic regression analysis of factors associated with ever seeking medical advice for change in bowel habit

	**OR (95% CI)**	***p* value**
Annual household income before tax ($)		
<= 39, 999	1	
$40, 000 - $69, 999	.36 (.13, .95)	.038
> = $70, 000	.29 (.10, .86)	.027
BMI		
< 18.5	-	
18.5 – 25	1	
> 25	.12 (.03, .59)	.009
Family history of CRC discussed with doctor		
Never discussed	1	
Discussed/informed of ‘increased risk’	5.68 (1.72, 18.75)	.004
Discussed/not informed of increased ‘risk’	2.90 (1.06, 7.94)	.038

OR, odds ratio; CI, confidence interval.

Of the 195 respondents ever experiencing a change in bowel habit persisting longer than two weeks, 37% (72/195) of persons had experienced this symptom for the first time in the previous five years and consulted a doctor. For this group, 63% of respondents respectively sought medical advice within four weeks. Multiple regression modelling indicated that early medical advice seeking (< 4 weeks) was significantly associated with persons who had: identified as a prompting factor for medical consultation - “Symptom didn’t go away”; fewer GP visits per year; and discussed their family history of CRC with a doctor and were notified of a possible ‘increased risk’ (See Table 5).

**Table 5:** Multiple logistic regression analysis of factors associated with early medical advice seeking for change in bowel habit

	**OR (95%CI)**	***p* value**
Every increase in GP visits per year	.52 (.31, .88)	.014
Prompt for medical consultation		
‘Symptom didn’t go away’	5.75 (1.42, 23.24)	.014
Family history of CRC discussed with doctor		
Never discussed	1	
Discussed/informed of ‘increased risk’	6.37 (1.04, 38.92)	.045
Discussed/not informed of increased ‘risk’	3.73 (.82, 16.97)	.089

OR, odds ratio; CI, confidence interval.

## Discussion

To our knowledge this study is the first to assess factors associated with *early* medical advice seeking behaviour for primary symptoms potentially indicating CRC. Previous studies have generally focused on rectal bleeding and the reasons for non-consultation or delay greater than three months [6,7,43]. For change in bowel habit, the current investigation of early medical advice seeking is timely given that research to date has generally examined factors associated with the decision to consult, rather than early medical advice seeking behaviour. 

### Failure to seek medical advice

The present study has highlighted that for both primary symptoms approximately one in five persons who had experienced symptoms had never consulted a doctor at any stage in their lifetime. A recent Australian population-based study of persons aged over 18 years indicated that 69% of respondents experiencing rectal bleeding had not presented to their physician in the previous year [58]. Other community-based studies have indicated that for persons aged over 40 years, approximately one-third either fail to seek, or delay (> 3 months) seeking medical advice for rectal bleeding [6,43]. A community based-study conducted in the United States identified that 86% of respondents experiencing rectal bleeding had failed to seek medical care within the prior year [59]. UK based studies have also indicated a high rate of non-consultation, between 59%-82% of respondents experiencing rectal bleeding [44,60]. For change in bowel habit, the literature is less developed. A UK study indicated that 76% of persons experiencing lower gastrointestinal symptoms (including functional bowel disorders) had failed to ever consult a doctor [41].

### Public health gain: decreasing CRC patients admitted to acute settings

At present, an alarming rate of 20-40% of all CRC cases present as a medical emergency [28,35,61]. Previous studies have indicated that symptomatic persons presenting to emergency departments report a median symptom duration of 3 months prior to admission, without necessarily presenting at an earlier point of intervention to primary care [33,62]. For cases presenting to emergency departments, the mortality rate is higher [35] and the cancer specific five year-survival rate is lower [36-38] which indicates that a significant public health gain is achievable if cases are identified and appropriately investigated within the primary health care setting [63]. Further, it is conceivable that improvements in earlier recognition of symptoms and immediate presentation to primary care could reduce the number of CRC cases requiring acute management options e.g. surgery for bowel obstruction. In theory, some emergency presentations should be prevented yet are not [33]. There is considerable room for improvement, with a significant proportion of community members (approximately 20% across both symptoms) never seeking medical advice for either potential symptom of CRC.

### Factors associated with ever seeking medical advice

Male persons were significantly more likely to seek medical advice for rectal bleeding. This finding is inconsistent with previous literature that has indicated males are less likely to present for medical care across a wide-trajectory of health issues [64,65]. Nonetheless, previous community-based studies relating to medical consultation for bowel related symptoms have generally indicated no gender difference [6,66]. Further, a systematic review of delay in diagnosis of CRC highlighted that sex had no impact on presentation times [67]. Future exploration of the barriers to help seeking for rectal bleeding among female persons and addressing such behaviours in public awareness campaigns may assist in improving overall consultation rates.

The present study identified that persons experiencing rectal bleeding who had ever received screening advice from a doctor or other health professional were significantly more likely to have ever sought medical advice. For this finding, the exact temporal sequencing of events was not ascertained, making extrapolation of exact cause and effect difficult. Intuitively persons may have experienced rectal bleeding, consulted a doctor and received screening advice after symptom episode. Alternatively persons may have received screening advice prior to symptom episode, with recollection of such a conversation prompting the increased likelihood of medical consultation. This temporal sequencing issue also relates to our finding that discussion of family history, regardless of whether the respondent was informed of increased risk, resulted in increased likelihood of ever consulting a doctor for change in bowel habit. Future research is required that clarifies the sequence and timing of such events.

Persons experiencing change in bowel habit with a lower household income were found to be significantly more likely to ever seek medical advice compared to persons with higher household income. In contrast, other community-based studies have found no relationship between socio-economic status and help seeking behaviour for rectal bleeding [42]. Similarly, no relationship between socio-economic status and help-seeking behaviour has been identified in relation to other cancer related symptoms [42,68]. Given the scant literature examining change in bowel habit and earlier presentation time, further investigation of broader socio-economic constructs effect on help-seeking behaviour is required.

### Factors associated with early medical advice seeking

The current study indicated that persons with private health coverage were significantly more likely to seek early medical advice for rectal bleeding. Such a finding is not surprising, given that persons without health insurance are known to have limited access to medical care [69] and poorer health outcomes [70,71] compared to privately insured persons. For this group it is proposed that increased morbidity and mortality of CRC is a result of restricted access to medical and surgical care [70,72]. In relation to CRC, health insurance status heavily influences access to care, screening and long-term outcomes [70,72,73]. Previous research has indicated that persons without health insurance are more likely to present with advanced cancer [73]. For CRC, uninsured and Medicaid populations have been found to be at greater risk of developing post-operative complications and in-hospital mortality compared to those privately insured [72]. More recent research also highlights longer pre- and post-presentation times for CRC patients without private health insurance [27]. Restricted access to health care or more concerning, lack of any medical advice seeking for those without private health insurance raises significant issues relating to possible delayed diagnosis, worse overall health, and advanced disease progression.

### Triggers of early medical advice seeking

Previous community and population-based studies have identified that perceived symptom seriousness is an important factor in eliciting medical consultation for rectal bleeding [44,45]. Studies that have examined retrospective recall of cancer patients have commonly identified that failure to recognise symptom seriousness is a significant factor associated with patient delay [74]. Previous population-based studies have indicated that failure to consult or delay (> 3 months) in seeking medical advice for rectal bleeding is due to an underestimation of symptom seriousness [6,43]. A similar finding has been demonstrated for patients recruited in the general practitioner setting [44]. The current study indicated that persons perceiving their symptom as serious were more likely to see a doctor at an earlier time point (< 2 weeks). This finding suggests that perception of symptom seriousness is not just an important factor for medical consultation but also contributes to earlier presentation time. Intuitively future health messages directed at the at-risk community must articulate the serious nature of primary symptoms of CRC and the importance of early medical advice seeking for improved health outcomes.

The current research also indicated that persons indicating that their trigger for medical consultation was “Opportunity to talk during visit for other reason” were more likely to not seek earlier medical advice. Such a finding suggests a person’s willingness to ‘sit on symptoms’ until another health issue or alternate reason for medical consultation arises. Encouragingly, current health messages worldwide are targeting this sub-group of persons with the “Don’t sit on your symptoms” campaign [75,76]. In relation to change in bowel habit, persons identifying “Symptom didn’t go away” as a trigger for medical consultation were more likely to consult at an earlier time point (< four weeks) compared to persons not identifying with this trigger.

### The need for improved medical advice seeking behaviour in the primary care setting

The study results suggest that there may be room for improvement in the identification of symptomatic patients in the primary care setting. General Practitioners (GPs) are in an ideal position to systematically offer information to patients with 88% of persons presenting to a GP annually, 30% aged 65 years or older [77]. Routine health screening relating to bowel related health may encourage earlier identification of symptoms. The acceptability and feasibility of systematic assessment of ovarian symptoms during GP practice visits is currently being assessed in the general practice setting [78]. A similar mechanism could be incorporated to monitor for potential symptom indicators of CRC, with particular attention on those subgroups who are less likely to seek prompt medical advice for symptoms.

### Study limitations

This study includes some limitations that should be considered when interpreting study results. The main limitation is a reliance on self-reported recall with no objective verification of symptom episode and time taken to seek medical advice. It is possible that recall bias may have affected some reports given that respondents were asked to report on circumstances that had occurred up to five years previously. To enhance respondents’ recall of the time taken to seek medical advice for primary symptoms of CRC, analysis was restricted to persons who had experienced their first symptom episodes in the previous five years and had consulted a doctor during this timeframe. This technique was adopted, as in other previous studies [6 43], to reduce the influence of potential recall bias. Nonetheless, this inherent limitation of studies using retrospective self report may be improved in the future through the adoption of a shorter recall period from symptom onset. Further, it should be considered that a conservative cut-point was used to denote early medical advice seeking for rectal bleeding (< 2 weeks) and change in bowel habit (< 4 weeks). Clinical practice guidelines in Australia and the United Kingdom (UK) do not specify a timeframe for at-risk persons (aged 50 years or over) to seek medical advice for primary symptoms of CRC [79,80]. In relation to rectal bleeding, at-risk persons are encouraged to seek prompt medical advice following symptom episode [79,80]. In relation to change in bowel habit, Australian guidelines encourage at-risk persons to seek medical advice but do not specify an exact time period [79]. However, guidelines in the UK do specify that persons 60 years or over experiencing changed bowel habit for longer than 6 weeks with no other anal symptoms be urgently referred [80]. Research on the predictive value of symptoms and patient delays effect on staging and prognosis of CRC should be used to inform any future amendments to guidelines or public health messages relating to “prompt” medical advice seeking behaviour. Finally, it must be noted that the relationship between the timing of discussions about family history of CRC with a doctor and provision of screening advice with symptom onset was not clearly delineated in the survey. Nonetheless, it is reasonable to assume that where health care providers have previously raised the issue of CRC, patients may subsequently feel more open about discussing potential symptoms and realise the importance of discussing symptoms.

## Conclusions

The high rate of failure to ever seek medical advice for primary symptoms potentially indicating CRC is an issue requiring public health action. Education about the seriousness of symptoms, particularly rectal bleeding and change in bowel habit in people over 50 years, and the need for early medical advice is required. Study results suggest the need for targeting of specific sub-groups in future public health messages encouraging prompt medical advice seeking. Importantly, patient delay in seeking medical advice is a modifiable factor that must be addressed if the burden of illness associated with CRC is to be reduced. Interventions within the primary health care setting are an important starting point to reach this critical endpoint.

## Competing interests

The authors declare that they have no competing interests.

## Authors’ contribution

Authors (RC, CP, RSF) made substantial contributions to the conception and design of the study. Data analysis was conducted by RC. All authors’ (RC, CP, RSF, FM, JA, MM) made substantial contributions to interpretation of data, drafting and editing of the manuscript and have given their approval of the manuscript for publication. All authors read and approved the final manuscript.

## Pre-publication history

The pre-publication history for this paper can be accessed here:

http://www.biomedcentral.com/1471-230X/12/100/prepub

## Supplementary Material

Additional file 1 Early detection of bowel cancer study. Click here for file

Additional file 2 **Simple logistic regression analyses of the factors associated with ever seeking medical advice for rectal bleeding and change in bowel habit.** Simple logistic regression analyses of socio-demographic, clinical and psychosocial factors association with early medical advice seeking. Click here for file
