# Supplementary material for: Factors associated with consultation behaviour for primary symptoms potentially indicating colorectal cancer: A cross-sectional study on response to symptoms
Source: BMC Gastroenterol. 2012 Aug 3;12:100. doi: 10.1186/1471-230X-12-100 (PMC3503829; doi:10.1186/1471-230X-12-100)
Supplement: Additional file 1 — Early detection of bowel cancer study. [file 1471-230X-12-100-S1.pdf]

Early Detection of Bowel Cancer Study

Hunter Community Study
Researching Health & Lifestyle in the Hunter Region

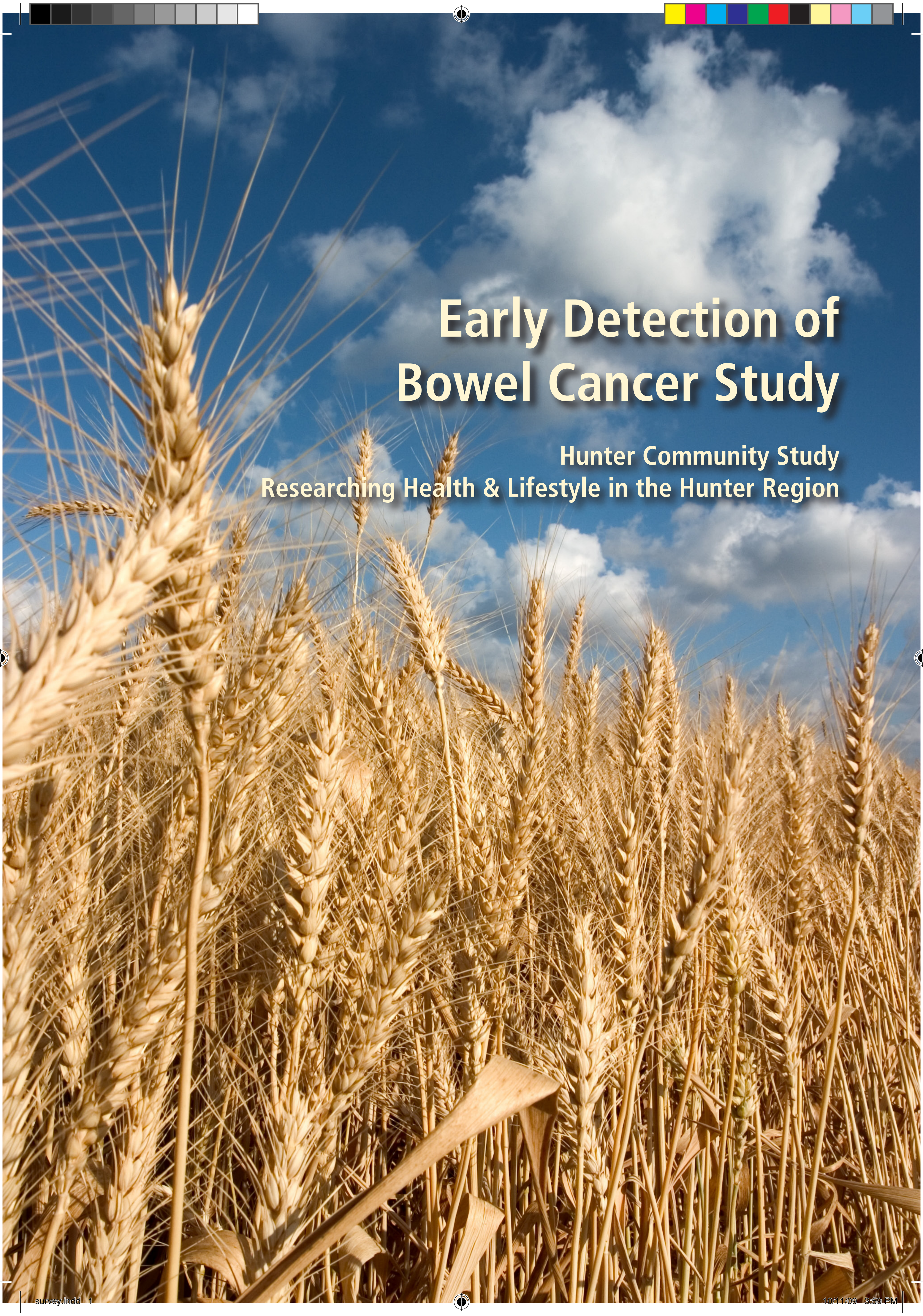

survey.indd 1 10/11/09 3:59 PM

hunter community study

Researching Health and Lifestyle in the Hunter Region

Professor John Attia • email hcs@newcastle.edu.au

All of the questions in this survey are about bowel cancer. They ask about symptoms, visiting the doctor, family history of bowel cancer and screening tests.

Please circle the number or letter that best describes your answer to each question. Some questions will require you to record numbers.

Please answer every question.

There are no right or wrong answers. The survey will take around 15 minutes to complete.

When you have completed all sections of the survey, simply put the survey in the reply-paid envelope provided and post it back within the next 7 days. No postage stamp is needed.

The return of your completed questionnaire will be taken as an indication of your voluntary consent to participate in this study.

If you have any questions or concerns about the study please do not hesitate to contact Dr Chris Paul (Study co ordinator) by telephone on 02 49 138143 or by email chris.paul@newcastle.edu.au

THANKS FOR YOUR TIME

If you have any concerns or complaints about the conduct of the study, you may contact:

Dr Nicole Gerrand
Professional Officer
Hunter New England Human Research Ethics Committee
Hunter New England Health
Locked Bag 1
New Lambton NSW 2305
Phone: (02) 49214950
Nicole.Gerrand@hnehealth.nsw.gov.au

HMRI
Hunter Medical Research Institute

In partnership with our community

THE UNIVERSITY OF NEWCASTLE AUSTRALIA | HUNTER NEW ENGLAND NSW HEALTH

**Q1**. Have you ever been told that you have a bowel related condition e.g. Inflammatory bowel disease - Ulcerative colitis or Crohn's disease?	1. **Yes**<br>2. **No**
**The following questions are about blood in your bowel motions**	
**Q2**. Have you ever noticed blood in your bowel motions, in the toilet bowl or on the toilet paper?	1. **Yes** ⇨ Go to Question 3<br>2. **No** ⇨ Go to Question 2a
**Q2a**. If you noticed blood or black material in a bowel motion how soon would you contact your doctor to make an appointment?	1. **Less than 1 week**<br>2. **More than 1 week but less than 4 weeks**<br>3. **More than 4 weeks but less than 8 weeks**<br>4. **More than 8 weeks but less than 12 weeks**<br>5. **More than 3 months but less than 12 months**<br>6. **More than 12 months**<br>7. **Never**<br>⇨ Go to Question 15
**Q3**. Have you ever seen a doctor about blood in your bowel motions, in the toilet bowel or on the toilet paper?	1. **Yes** ⇨ Go to Question 4<br>2. **No** ⇨ Go to Question 3a
**Q3a**. If you noticed blood or black material in a bowel motion ('sign of rectal bleeding') how soon would you contact your doctor to make an appointment?	1. **Less than 1 week**<br>2. **More than 1 week but less than 4 weeks**<br>3. **More than 4 weeks but less than 8 weeks**<br>4. **More than 8 weeks but less than 12 weeks**<br>5. **More than 3 months but less than 12 months**<br>6. **More than 12 months**<br>7. **Never**<br>⇨ Go to Question 14
**Q4**. Did you first notice blood in your bowel motions, in the toilet bowl or on the toilet paper in the last 5 years?	1. **Yes** ⇨ Go to Question 5<br>2. **No** ⇨ Go to Question 4a

**Q4a**. If you noticed blood or black material in a bowel motion ('sign of rectal bleeding') how soon would you contact your doctor to make an appointment?	**1. Less than 1 week**<br>**2. More than 1 week but less than 4 weeks**<br>**3. More than 4 weeks but less than 8 weeks**<br>**4. More than 8 weeks but less than 12 weeks**<br>**5. More than 3 months but less than 12 months**<br>**6. More than 12 months**<br>**7. Never**<br>⇨ Go to Question 15
**Q5**. How long ago did you notice blood in your bowel motions for the first time?<br>*(Please specify the amount of time)*	**a. _____________ Days**<br>**b. _____________ Weeks**<br>**c. _____________ Months**<br>**d. _____________ Years**
**Q5a**. Did you see a doctor about it?	**1. Yes**<br>**2. No** ⇨ Go to Question 14
**Q6**. How long after you first noticed blood in your bowel motions did you see a doctor about it?<br>*(Please specify the amount of time)*	**a. _____________ Days**<br>**b. _____________ Weeks**<br>**c. _____________ Months**<br>**d. _____________ Years**
**Q7**. What is the main reason you did not seek medical advice about blood in your bowel motions sooner?<br>*(Please circle only one response)*	**1. I consulted a doctor within one week**<br>**2. Thought it wasn't serious**<br>**3. Cleared up itself**<br>**4. Thought it was haemorrhoids/piles**<br>**5. Decided to wait and see**<br>**6. Seeing a doctor would be unpleasant / embarrassing**<br>**7. Worried or scared it might be serious**<br>**8. No faith in doctors / doctor couldn't do anything**<br>**9. Couldn't afford doctor visit**<br>**10. Did not want to waste doctor's time**<br>**11. Not confident in discussing symptom / doctor hard to talk to**<br>**12. No time/busy/other things to think about**<br>**13. Difficulty making an appointment or getting transport**<br>**14. Didn't want to worry family/friends**<br>**15. Can't remember**<br>**16. Other (please specify____________________________)**

**Q8**. Where did you notice this blood?<br>*(Please circle all that apply)*	a. Blood mixed with stool / poo<br>b. Blood on toilet paper<br>c. Blood in the toilet bowl
**Q9**. How would you describe the colour of the blood?	1. Bright red<br>2. Dark red<br>3. Black
**Q10**. How often was there blood in bowel motion?	1. Every time<br>2. More than half of the time but not all the time<br>3. About half of the time<br>4. Less than half the time but more than once<br>5. Only once<br>6. Not sure
**Q11**. How much did the amount of blood concern you?	1. High concern<br>2. Moderate concern<br>3. Minor concern<br>4. No concern at all
**Q12**. What in particular prompted you to consult a doctor about blood in your bowel motions?<br>*(Please circle all that apply)*	1. Thought the symptom was serious<br>2. Symptom didn't go away<br>3. Symptom was bad<br>4. Symptom got worse<br>5. Partner or family member suggested it<br>6. Advertisement about bowel cancer<br>7. Friend diagnosed with cancer<br>8. Family history of cancer<br>9. Opportunity to talk during doctor visit for other reason<br>10. Had check up or appointment already booked<br>11. Other (please specify______________________)

**Q13**. What did your doctor do when you mentioned the blood in your bowel movements?<br>*(Please circle all that apply)*	**1. Discussed symptoms**<br>**2. Advised to make dietary change e.g. higher fibre diet**<br>**3. Told to 'wait and see'**<br>**4. Digital rectal examination (DRE)/Physical examination**<br>**5. Referral to bowel specialist for screening test e.g. colonoscopy/sigmoidoscopy**<br>**6. Sent for test to detect blood in stool/poo e.g. Faecal Occult Blood Test (FOBT)**<br>**7. Blood test**<br>**8. Diagnosed with haemorrhoids/piles**<br>**9. Diagnosed with bowel related condition e.g. Irritable bowel syndrome, no further action taken**<br>⇨ Go to Question 15

**Q14**. What is the main reason you did not seek medical advice about blood in your bowel motions?<br>*(Please circle only one response)*	1. **Thought it wasn't serious** ⇨ Go to Question 14a<br>2. **Cleared up itself** ⇨ Go to question 14b<br>3. **Thought it was haemorrhoids/piles** ⇨ Go to Question 15<br>4. **Decided to wait and see** ⇨ Go to Question 15<br>5. **Seeing doctor would be unpleasant/embarrassing** ⇨ Go to Question 15<br>6. **Worried that the doctor might find something serious (scared)** ⇨ Go to Question 15<br>7. **Never see doctors/little faith in doctors** ⇨ Go to Question 15<br>8. **Thought doctor couldn't do anything** ⇨ Go to Question 15<br>9. **Couldn't afford doctor visit** ⇨ Go to Question 15<br>10. **Did not want to waste doctor's time** ⇨ Go to Question 15<br>11. **Not confident in discussing symptom** ⇨ Go to Question 15<br>12. **Doctor difficult to talk to** ⇨ Go to Question 15<br>13. **No time/busy/other things to worry about** ⇨ Go to Question 15<br>14. **Put it out of my mind – chose not to think about the symptom** ⇨ Go to Question 15<br>15. **Difficulty making an appointment** ⇨ Go to Question 15<br>16. **Didn't want to worry family/friends** ⇨ Go to Question 15<br>17. **Transport problems** ⇨ Go to Question 15<br>18. **Can't remember** ⇨ Go to Question 15<br>19. **Other (please specify________________________)**
**Q14a**. Which symptom(s) would be serious enough for you to see a doctor?<br>*(Please circle all that apply)*	a. **Black blood**<br>b. **Blood in the stool**<br>c. **Blood every time I passed bowel movements**<br>d. **Lots of blood**<br>⇨ Go to Question 15

survey.indd 7 10/11/09 4:00 PM

**Q14b**. How long did it take to clear up? *(Please specify the amount of time)*	**a.** ____________ **Days**<br>**b.** ____________ **Weeks**<br>**c.** ____________ **Months**<br>**d.** ____________ **Years**
**The following questions are about a change in normal bowel habits**	
**Q15**. Have you ever noticed a persistent change in your normal bowel habits that lasted longer than two weeks? This may include looser bowel motion/ needing to go to the toilet more than usual (diarrhoea) or an inability to pass bowel motions (constipation).	**1. Yes** ⇨ Go to Question 16<br>**2. No** ⇨ Go to Question 15a
**Q15a**. If you noticed a persistent change in your bowel habits lasting longer than two weeks, how soon would you contact your doctor to make an appointment?	**1. Less than 1 week**<br>**2. More than 1 week but less than 4 weeks**<br>**3. More than 4 weeks but less than 8 weeks**<br>**4. More than 8 weeks but less than 12 weeks**<br>**5. More than 3 months but less than 12 months**<br>**6. More than 12 months**<br>**7. Never**<br>⇨ Go to Question 28
**Q16**. Have you ever seen a doctor about a persistent change in your normal bowel habits that lasted longer than two weeks?	**1. Yes** ⇨ Go to Question 17<br>**2. No** ⇨ Go to Question 16a
Q16a. If you noticed a persistent change in your bowel habits lasting longer than two weeks, how soon would you contact your doctor to make an appointment?	**1. Less than 1 week**<br>**2. More than 1 week but less than 4 weeks**<br>**3. More than 4 weeks but less than 8 weeks**<br>**4. More than 8 weeks but less than 12 weeks**<br>**5. More than 3 months but less than 12 months**<br>**6. More than 12 months**<br>**7. Never**<br>⇨ Go to Question 27
**Q17**. Did you first notice a persistent change in your bowel habits that lasted longer than two weeks in the last 5 years?	**1. Yes** ⇨ Go to Question 18<br>**2. No** ⇨ Go to Question 17a

**Q17a**. If you noticed a persistent change in your bowel habits lasting longer than two weeks, how soon would you contact your doctor about it to make an appointment?	1. **Less than 1 week**<br>2. **More than 1 week but less than 4 weeks**<br>3. **More than 4 weeks but less than 8 weeks**<br>4. **More than 8 weeks but less than 12 weeks**<br>5. **More than 3 months but less than 12 months**<br>6. **More than 12 months**<br>7. **Never** ⇨ Go to Question 28
**Q18**. How long ago did you notice a persistent change in your bowel habits for the first time?<br>*(Please specify the amount of time)*	a. ____________ **Days**<br>b. ____________ **Weeks**<br>c. ____________ **Months**<br>d. ____________ **Years**
**Q19**. What type of irregular bowel movements did you experience?<br>*(Please circle all that apply)*	a. **Diarrhoea**<br>b. **Constipation**<br>c. **Other (please specify** __________________________ **)**
**Q19a**. Did you see a doctor about it?	1. **Yes**<br>2. **No** ⇨ Go to Question 27
**Q20**. How long after you first noticed a persistent change in your bowel habits did you see a doctor about it?<br>*(Please specify the amount of time)*	a. ____________ **Days**<br>b. ____________ **Weeks**<br>c. ____________ **Months**<br>d. ____________ **Years**

**Q21**. What is the main reason you did not seek medical advice about a persistent change in your bowel habits sooner?<br>*(Please circle only one response)*	1. **I consulted a doctor within one week** ⇨ Go to Question 22<br>2. **Thought it wasn't serious** ⇨ Go to Question 22<br>3. **Cleared up itself** ⇨ Go to Question 21a<br>4. **Thought it was haemorrhoids/piles** ⇨ Go to Question 22<br>5. **Decided to wait and see** ⇨ Go to Question 22<br>6. **Seeing doctor would be unpleasant/embarrassing** ⇨ Go to Question 22<br>7. **Worried or scared it might be serious** ⇨ Go to Question 22<br>8. **No faith in doctors/doctor couldn't do anything** ⇨ Go to Question 22<br>9. **Couldn't afford doctor visit** ⇨ Go to Question 22<br>10. **Did not want to waste doctor's time** ⇨ Go to Question 22<br>11. **Not confident in discussing symptom/doctor hard to talk to** ⇨ Go to Question 22<br>12. **No time/busy/other things to think about** ⇨ Go to Question 22<br>13. **Difficulty making an appointment or getting transport** ⇨ Go to Question 22<br>14. **Didn't want to worry family/friends** ⇨ Go to Question 22<br>15. **Can't remember** ⇨ Go to Question 22<br>16. **Other (please specify________________________)** ⇨ Go to Question 22
**Q21a**. How long did it take to clear up?<br>*(Please specify the amount of time)*	**a.** ____________ **Days**<br>**b.** ____________ **Weeks**<br>**c.** ____________ **Months**<br>**d.** ____________ **Years**
**Q22**. How long did the change in bowel habits persist?<br>*(Please specify the amount of time)*	**a.** ____________ **Days**<br>**b.** ____________ **Weeks**<br>**c.** ____________ **Months**<br>**d.** ____________ **Years**

survey.indd 10 10/11/09 4:00 PM

**Q23**. Please rate how uncomfortable or painful this diarrhoea/ constipation was?	1. **No discomfort**<br>2. **Mild discomfort**<br>3. **Mild pain**<br>4. **Moderate pain**<br>5. **Intense pain**
**Q24**. How often did you experience diarrhoea/ constipation when passing bowel motion?	1. **Every time**<br>2. **More than half of the time but not all the time**<br>3. **About half of the time**<br>4. **Less than half the time but more than once**<br>5. **Only once**<br>6. **Not sure**
**Q25**. What in particular prompted you to consult a doctor about a persistent change in your bowel habits?<br>*(Please circle all that apply)*	1. **Thought the symptom was serious**<br>2. **Symptom didn't go away**<br>3. **Symptom was bad**<br>4. **Symptom got worse**<br>5. **Partner or family member suggested it**<br>6. **Advertisement about bowel cancer**<br>7. **Friend diagnosed with cancer**<br>8. **Family history of cancer**<br>9. **Opportunity to talk during doctor visit for other reason**<br>10. **Had check up or appointment already booked**<br>11. **Other (please specify______________________________)**
**Q26**. What did your doctor do when you mentioned a persistent change in your bowel habits?<br>*(Please circle all that apply)*	a. **Discussed symptoms**<br>b. **Advised to make dietary change e.g. higher fibre diet**<br>c. **Told to 'wait and see'**<br>d. **Digital rectal examination (DRE)/Physical Examination**<br>e. **Referral to bowel specialist for screening test e.g. colonoscopy/sigmoidoscopy**<br>f. **Sent for test to detect blood in stool/poo e.g. Faecal Occult Blood Test (FOBT)**<br>g. **Blood test**<br>h. **Diagnosed with Haemorrhoids/piles**<br>i. **Diagnosed with bowel related condition e.g. Irritable bowel syndrome, no further action taken.**<br>⇨ Go to Question 28

survey.indd 11 10/11/09 4:00 PM

**Q27**. What is the main reason you did not seek medical advice about a persistent change in your bowel habits?<br>*(Please circle only one response)*	1. **Thought it wasn't serious** ⇨ Go to Question 28<br>2. **Cleared up itself** ⇨ Go to Question 27a<br>3. **Thought it was haemorrhoids/piles** ⇨ Go to Question 28<br>4. **Decided to wait and see** ⇨ Go to Question 28<br>5. **Seeing doctor would be unpleasant/embarrassing** ⇨ Go to Question 28<br>6. **Worried that the doctor might find something serious (scared)** ⇨ Go to Question 28<br>7. **Never see doctors/little faith in doctors** ⇨ Go to Question 28<br>8. **Thought doctor couldn't do anything** ⇨ Go to Question 28<br>9. **Couldn't afford doctor visit** ⇨ Go to Question 28<br>10. **Did not want to waste doctor's time** ⇨ Go to Question 28<br>11. **Not confident in discussing symptom** ⇨ Go to Question 28<br>12. **Doctor difficult to talk to** ⇨ Go to Question 28<br>13. **No time/busy/other things to worry about** ⇨ Go to Question 28<br>14. **Put it out of my mind – chose not to think about the symptom** ⇨ Go to Question 28<br>15. **Difficulty making an appointment** ⇨ Go to Question 28<br>16. **Didn't want to worry family/friends** ⇨ Go to Question 28<br>17. **Transport problems** ⇨ Go to Question 28<br>18. **Can't remember** ⇨ Go to Question 28<br>19. **Other (please specify______________________________)**
**Q27a**. How long did it take to clear up?<br>*(Please specify the amount of time*	a. _____________ **Days**<br>b. _____________ **Weeks**<br>c. _____________ **Months**<br>d. _____________ **Years**

HMRI
Hunter Medical Research Institute

In partnership with our community

THE UNIVERSITY OF NEWCASTLE AUSTRALIA | HUNTER NEW ENGLAND NSW HEALTH

survey.indd 12 10/11/09 4:00 PM

Q28. If you noticed any of the following, how soon would you contact your doctor to make an appointment? Record the answer to each question that best applies to you by circling:

	Less than a week	More than 1 week but less than 4 weeks	More than 4 weeks but less than 8 weeks	More than 8 weeks but less than 12 weeks	More than 3 months but less than 12 months	More than 12 months	Never
a. Unexplained weight loss	1	2	3	4	5	6	7
b. Unexplained tiredness or weakness	1	2	3	4	5	6	7
c. Abdominal pain	1	2	3	4	5	6	7
d. Loss of Appetite	1	2	3	4	5	6	7

The following questions are about appropriate bowel cancer screening

The following questions are about whether any of your relatives have been diagnosed with bowel cancer. Bowel cancer, also called colorectal cancer, is cancer in the large bowel or large intestine, it includes both colon and rectal cancer but not anal cancer.

Q29. How many of your close relatives (mother, father, brother, sister or child) have ever been diagnosed with bowel cancer?

a. **None** ⇨ Go to Question 31
b. **One**
c. **Two**
d. **Three or more**

Q30. How many of these relative(s) were diagnosed at the following ages?
(Please write number of relatives diagnosed in each age category)

1. _____**50 or less**
2. _____**Between 51 and 55**
3. _____**56 or over**
4. _____**Don't know**

In partnership with our community

HMRI Hunter Medical Research Institute | The University of Newcastle Australia | Hunter New England NSW Health

**Q31**. Have any of your second-degree relatives (grandparents, uncles, aunts, nephews, nieces or half-siblings) ever been diagnosed with bowel cancer? This includes only biological/blood-relatives, not those related to you through marriage.<br>*(Please select all that apply)*	**a. None of these relatives have ever been diagnosed with bowel cancer** ⇨ Go to Question 33<br>**b. Yes, mother's mother**<br>**c. Yes, father's mother**<br>**d. Yes, mother's father**<br>**e. Yes, father's father**<br>**f. Yes, mother's sister**<br>**g. Yes, father's sister**<br>**h. Yes, mother's brother**<br>**i. Yes, father's brother**<br>**j. Yes, nephew**<br>**k. Yes, niece**<br>**l. Yes, grandchild**<br>**m. Yes, half-sibling (mother's side)**<br>**n. Yes, half-sibling (father's side)**
**Q32**. How many of these relative(s) were diagnosed at the following ages?<br>*(Please write number of relatives diagnosed in each age category)*	**1. ____50 or less**<br>**2. ____Between 51 and 55**<br>**3. ____56 or over**<br>**4. ____Don't know**
**Q33**. Has your mother, father, brother, sister, child, grandparent, uncle, aunt, nephew, niece or half-sibling ever been diagnosed with any of the following cancers: endometrium, ovary, stomach, renal pelvis, ureter, biliary tract or brain.	**1. Yes**<br>**2. No**<br>**3. Don't know**
**The next questions are about *screening tests* you may have had for bowel cancer.**	
**Q34.** Have you ever had an FOBT, FIT or iFOBT? These tests faecal occult blood test (FOBT), faecal immunochemical test (FIT) and immunochemical faecal occult blood test (iFOBT) involve you providing samples of faeces or poo. The samples would have been sent to a laboratory to test for tiny amounts of blood.	**1. Yes**<br>**2. No** ⇨ Go to Question 37<br>**3. Not Sure** ⇨ Go to Question 37

**Q35**. How long ago was your last FOBT, FIT or iFOBT?	1. **Less than a month ago**<br>2. **One month to less than twelve months ago**<br>3. **Twelve months to less than two years ago**<br>4. **Two years to less than five years ago**<br>5. **Five years or longer ago**<br>6. **Don't know/not sure**
**Q36**. Did you have your last FOBT, FIT or iFOBT because you had a symptom?	1. **Yes**<br>2. **No**
**Q37**. Have you ever had a colonoscopy? This is usually a day procedure in hospital where the inside of your colon is examined while you are sedated.	1. **Yes**<br>2. **No** ⇨ Go to Question 40<br>3. **Not Sure** ⇨ Go to Question 40
**Q38**. How long ago was your most recent colonoscopy?	1. **Less than a month ago**<br>2. **One month to less than twelve months ago**<br>3. **Twelve months to less than two years ago**<br>4. **Two years to less than five years ago**<br>5. **Five years or longer ago**<br>6. **Don't know/not sure**
**Q39**. Did you have your most recent colonoscopy because you had a symptom?	1. **Yes**<br>2. **No**
**Q40**. Have you ever had a sigmoidoscopy? In this procedure only the rectum and lower part of the colon are examined. This is a short procedure which lasts about 5-10 minutes. Sedation is not usually required and you can usually go home straight after the procedure.	1. **Yes**<br>2. **No** ⇨ Go to Question 43<br>3. **Not Sure** ⇨ Go to Question 43
**Q41**. How long ago was your most recent sigmoidoscopy?	1. **Less than a month ago**<br>2. **One month to less than twelve months ago**<br>3. **Twelve months to less than two years ago**<br>4. **Two years to less than five years ago**<br>5. **Five years or longer ago**
**Q42**. Did you have your most recent sigmoidoscopy because you had a symptom?	1. **Yes**<br>2. **No**

The following questions ask about risk

You may or may not be aware that an individual's risk of developing bowel cancer increases with the number of relatives diagnosed in a family. Sometimes doctors may ask questions about your family history of colorectal cancer and give advice on screening for you and your relatives.

**Q43**. Has any health professional e.g. your doctor, ever asked if you have a family history of bowel cancer?	**1. Yes**<br>**2. No ⇨ Go to Question 45**
**Q44**. Did this person discuss whether there was a possible 'increased risk' of bowel cancer for you or your family members?	**1. Yes**<br>**2. No**
**Q45**. Has any health professional suggested that you or your relatives should do any of the following:<br>*Please choose all that apply.*	**a. Start having screening tests for bowel cancer**<br>**b. Talk to their doctor about screening tests**<br>**c. No advice has ever been given**<br>**d. Take other action related to bowel cancer**<br>**(please specify ____________________)**

The following questions ask about internet use

We are interested in finding out whether people of various ages would be interested in answering surveys over the internet.

**Q46**. Do you use the internet?<br>*(Please circle all that apply)*	**a. Yes, at home**<br>**b. Yes, at work**<br>**c. Yes, at library or café**<br>**d. No, not at all**
**Q47**. Do you have access to email?<br>*(Please circle all that apply)*	**a. Yes, at home**<br>**b. Yes, at work**<br>**c. Yes, at library or café**<br>**d. No, not at all**

If you have any questions, concerns or worries about cancer after doing this survey, we encourage you to discuss matters with your doctor or contact the Cancer Council Helpline, on 13 11 20.

You have now finished this survey.
Thank you for your time.
Please return the completed questionnaire to the Hunter Community Study in the reply-paid envelope provided.

survey.indd 16 10/11/09 4:00 PM
