# Supplementary material for: Factors associated with consultation behaviour for primary symptoms potentially indicating colorectal cancer: A cross-sectional study on response to symptoms
Source: BMC Gastroenterol. 2012 Aug 3;12:100. doi: 10.1186/1471-230X-12-100 (PMC3503829; doi:10.1186/1471-230X-12-100)
Supplement: Additional file 2 — Simple logistic regression analyses of the factors associated with ever seeking medical advice for rectal bleeding and change in bowel habit. Simple logistic regression analyses of socio-demographic, clinical and psychosocial factors association with early medical advice seeking. [file 1471-230X-12-100-S2.docx]

**Additional file 1**

**Table 1.**

Simple logistic regression analyses of the factors associated with ever seeking medical advice for rectal bleeding and change in bowel habit.

| Primary symptom | Rectal Bleeding | | Change in bowel habit | |
| --- | --- | --- | --- | --- |
|  | OR (95% CI) | *p* value | OR (95% CI) | *p* value |
| Socio-demographic characteristics |  |  |  |  |
| Gender |  |  |  |  |
| Female | 1 |  |  |  |
| Male | .58 (.33, 1.03) | **.063** | 1.90 (.93, 3.86) | **.078** |
| Age (years) |  |  |  |  |
| 56-64 | 1 |  | 1 |  |
| 65-74 | 1.82 (.94, 3.49) | **.073** | 1.71(.70, 4.19) | **.238** |
| 75-88 | 1.12 (.52, 2.40) | .774 | 1.35 (.55, 3.36) | .512 |
| Marital status |  |  |  |  |
| Married/ Living with partner | 1 |  | 1 |  |
| Never married/ Widowed/ Divorced or separated | 1.85 (.89, 3.85) | **.098** | 1.46 (.66, 3.25) | .354 |
| Education |  |  |  |  |
| Secondary schooling (not-completed) | 1 |  | 1 |  |
| Secondary schooling (completed) | 1.17 (.49, 2.74) | .723 | 1.41 (.48, 4.17) | .530 |
| Trade qualification or TAFE: | 1.42 (.60, 3.39) | .421 | 1.18 (.43, 3.27) | .748 |
| University or other tertiary study | .93 (.43, 2.00) | .848 | 1.53 (.59, 3.98) | .380 |
| Other or not applicable | 1.44 (.29, 7.22) | .65 | - |  |
| Household income before tax ($) |  |  |  |  |
| <= 39, 999 | 1 |  | 1 |  |
| 40, 000 – 69, 999 | 1.03 (.49, 2.16) | .935 | .44 (.19, 1.05) | **.064** |
| >= 70,000 | .57 (.28, 1.17) | **.128** | .41 (.16, 1.06) | **.065** |
| Country of birth |  |  |  |  |
| Australian | 1 |  | 1 |  |
| Other | .53 (.23, 1.21) | **.133** | .94 (.29, 3.05) | .925 |
| Retired |  |  |  |  |
| Yes | 1.64 (.93, 2.88) | **.088** | 1.12 (.55, 2.31) | .748 |
| No | 1 |  | 1 |  |
| Private health insurance |  |  |  |  |
| No coverage | 1 |  | 1 |  |
| Coverage | .76 (.37, 1.55) | .451 | .75 (.32, 1.78) | .514 |
| Drink days per month (Alcohol) | 1.00 (.97, 1.03) | .828 | .98 (.95, 1.01) | .259 |
| Smoke |  |  |  |  |
| Never | 1 |  | 1 |  |
| Ever | .84 (.47, 1.52) | .566 | .74 (.35, 1.54) | .417 |
| Now | .50 (.15, 1.70) | .266 | .34 (.05, 2.17) | .253 |
| Clinical characteristics |  |  |  |  |
| GP visits per year | 1.15 (.93, 1.43) | **.20** | 1.08 (.85, 1.37) | .522 |
| Previous Cancer (excluding CRC) |  |  |  |  |
| Yes | .83 (.43, 1.63) | .592 | .59 (.26, 1.32) | **.200** |
| No | 1 |  | 1 |  |
| First degree relative diagnosed CRC |  |  |  |  |
| Yes | 5.93 (1.40, 25.18) | **.016** | 2.02 (.66, 6.16) | **.217** |
| No | 1 |  | 1 |  |
| Ever discussed Family history of CRC with doctor |  |  |  |  |
| Never discussed | 1 |  | 1 |  |
| Discussed, informed of ‘increased risk’ | 5.70 (2.31, 14.08) | **.000** | 3.10 (1.17, 8.23) | **.023** |
| Discussed, not informed of ‘increased risk’ | 2.22 (1.09, 4.53) | **.028** | 2.02 (.82, 4.95) | **.125** |
| Screening advice ever given by doctor |  |  |  |  |
| Yes | 4.67 (2.13, 10.25) | **.000** | 1.23 (.57, 2.66) | .591 |
| No | 1 |  | 1 |  |
| BMI |  |  |  |  |
| < 18.5 | - |  | - |  |
| 18.5 - 25 | 1 |  | 1 |  |
| > 25 | .87 (.40, 1.90) | .729 | .28 (.08, .98) | **.046** |
| Comorbidity |  |  |  |  |
| Yes | 1.43 (.70, 2.94) | .323 | 1.53 (.59, 3.98) | .378 |
| No | 1 |  | 1 |  |
| Psychosocial characteristics |  |  |  |  |
| SF-36 (physical health) | .99 (.96, 1.02) | .642 | 1 (.97, 1.03) | .922 |
| K-10 (mental health) |  |  |  |  |
| Low or no risk (10–15) | 1 |  | 1 |  |
| Medium to high risk (16 +) | 1.28 (.66, 2.47) | .457 | .83 (.40, 1.73) | .627 |

*Variables with p* values < .25 included in multiple logistic regression model bolded

**Table 2.**

Simple logistic regression analyses of socio-demographic, clinical and psychosocial factors association with early medical advice seeking.

| Primary symptom | Rectal bleeding  (<2 weeks) | | Change in bowel habit  (< 4 weeks) | |
| --- | --- | --- | --- | --- |
|  | OR (95% CI) | *p* value | OR (95% CI) | *p* value |
| Socio-demographic characteristics |  |  |  |  |
| Gender |  |  |  |  |
| Female | 1 |  | 1 |  |
| Male | .55 (.23, 1.30) | **.177** | .54 (.19, 1.51) | **.244** |
| Age (years) |  |  |  |  |
| 56-64 | 1 |  | 1 |  |
| 65-74 | 2.05 (.80, 5.26) | **.136** | 1.18 (.39, 3.64) | .768 |
| 75-88 | 1.57 (.49, 5.04) | .443 | 1.14 (.31, 4.22) | .848 |
| Marital status |  |  |  |  |
| In a relationship | 1 |  | 1 |  |
| Not in relationship | .76 (.28, 2.09) | .601 | .98 (.34, 2.87) | .980 |
| Education |  |  |  |  |
| Secondary schooling (not-completed) | 1 |  | 1 |  |
| Secondary schooling (completed) | 1.25 (.34, 4.56) | .735 | .5 (.09, 2.80) | .431 |
| Trade qualification or TAFE: | 1.31 (.37, 4.58) | .670 | .71 (.15, 3.33) | .669 |
| University or other tertiary study | .97 (.28, 3.37) | .965 | 1.25 (.27, 5.76) | .775 |
| Other or not applicable | 2.33 (.21, 25.24) | .486 | .75 (.08, 6.46) | .794 |
| Household income before tax ($) |  |  |  |  |
| <= 39, 999 | 1 |  | 1 |  |
| 40, 000 – 69, 999 | 1.7 (.58, 4.99) | .334 | .75 (.21, 2.62) | .649 |
| >= 70,000 | 2.40 (.60, 9.50) | **.212** | 2.24 (.41, 12.16) | .350 |
| Country of birth |  |  |  |  |
| Australian | 1 |  | - |  |
| Other | 1.19 (.29, 4.93) | .704 |  |  |
| Retired |  |  |  |  |
| Yes | 1.07 (.44, 2.57) | .881 | 1.01 (.35, 2.94) | .980 |
| No | 1 |  | 1 |  |
| Private health insurance |  |  |  |  |
| No coverage | 1 |  | 1 |  |
| Coverage | 2.29 (.85, 6.20) | **.102** | .47 (.15, 1.44) | .189 |
| Drink days per month (Alcohol) | 1.03 (.99, 1.08) | **.090** | 1.01 (.96, 1.06) | .618 |
| Smoke |  |  |  |  |
| Never | 1 |  | 1 |  |
| Ever | .90 (.36, 2.34) | .828 | 2.84 (.97, 8.29) | **.056** |
| Now | - | - |  |  |
| Clinical characteristics |  |  |  |  |
| GP visits per year | 1.07 (.79, 1.47) | .628 | .72 (.50, 1.05) | **.088** |
| Previous Cancer (excluding CRC) |  |  |  |  |
| Yes | .88 (.29, 2.65) | .824 | 2.1 (.59, 7.41) | .249 |
| No | 1 |  | 1 |  |
| First degree relative diagnosed CRC |  |  |  |  |
| Yes | .44 (.15, 1.32) | **.146** | 2.8 (.70, 11.16) | **.144** |
| No | 1 |  | 1 |  |
| Ever discussed Family history of CRC with doctor |  |  |  |  |
| Never discussed | 1 |  | 1 |  |
| Discussed, informed of ‘increased risk’ | 1.71 (.56, 5.20) | .341 | 7.46 (1.70, 32.67) | **.008** |
| Discussed, not informed of ‘increased risk’ | .65 (.23, 1.82) | .415 | 3.2 (.96, 10.65) | **.058** |
| Screening advice ever given by doctor |  |  |  |  |
| Yes | 1.19 (.48, 2.96) | .705 | 1.23 (.41, 3.69) | .712 |
| No | 1 |  | 1 |  |
| BMI |  |  |  |  |
| < 18.5 |  |  |  |  |
| 18.5 - 25 | 1 |  | 1 |  |
| > 25 | .89 (.28, 2.85) | .845 | 1.41 (.34, 5.82) | .636 |
| Comorbidity |  |  |  |  |
| Yes | .33 (.07, 1.60) | **.170** | 1.41 (.34, 5.83) | .636 |
| No | 1 |  | 1 |  |
| Psychosocial characteristics |  |  |  |  |
| SF-36 (physical health) | 1.01 (.98, 1.06) | .347 | .98 (.94, 1.03) | .428 |
| K-10 (mental health) |  |  |  |  |
| Low or no risk (10–15) | 1 |  | 1 |  |
| Medium to high risk (16 +) | .35 (.14, .89) | **.028** | .69 (.25, 1.96) | .494 |
| Trigger for seeking medical advice |  |  |  |  |
| Thought the symptom was serious | 5.43 (1.87, 15.77) | **.002** | 2.22 (.55, 8.99) | .263 |
| Symptom didn’t go away | .98 (.35, 2.72) | .971 | 2.63 (.95, 7.27) | **.061** |
| Symptom was bad | .47 (.06, 3.54) | .469 | 1.80 (.17, 18.29) | .619 |
| Symptom got worse | .63(.13, 3.01) | .568 | .27 (.02, 3.18) | .301 |
| Partner or family member suggested it | .98 (.27, 3.53) | .979 | - |  |
| Advertisement about bowel cancer | 4.33 (.52, 36.25) | **.175** | 3.15 (.35, 28.70) | .307 |
| Friend diagnosed with cancer | .48 (.03, 8.00) | .613 | - |  |
| Family history of cancer | .30 (.09, .98) | **.046** | 4.66 (.54, 40.38) | **.162** |
| Opportunity to talk during doctor visit for other reason | .21 (.07, .58) | **.003** | .41 (.09, 1.69) | .219 |
| Had check up or appointment already booked | 2.58 (.29, 23.03) | .396 | .36 (.05, 2.30) | .279 |
| Symptom characteristics |  |  |  |  |
| Frequency |  |  |  |  |
| Only once to less than half the time | 1 |  | 1 |  |
| Half the time to every time | 1.56 (.59, 4.15) | .370 | .70 (.19, 2.52) | .592 |
| Not sure | .43 (.12, 1.46) | **.176** |  |  |
| Location of blood |  |  |  |  |
| Blood mixed with stool | .79 (.26, 2.40) | .677 | - |  |
| Blood on toilet paper | .84 (.29, 2.43) | .750 |  |  |
| Blood in the toilet bowl | .97 (.41, 2.29) | .955 |  |  |
| Concern |  |  |  |  |
| Moderate to high concern | 1 |  | - |  |
| None to minor concern | .33 (.13, .86) | **.022** |  |  |
| Type of bowel movement |  |  |  |  |
| Diarrhoea | - |  | .78 (.28, 2.16) | .634 |
| Constipation |  |  | .76 (.32, 2.30) | .761 |
| Discomfort/ pain |  |  |  |  |
| None to mild discomfort | - |  | 1 |  |
| Mild to intense pain |  |  | 1.50 (.53, 4.28) | .443 |

*Variables with p values < .25 included in multiple logistic regression model bolded*
